# Close Relationships Despite the Challenges: Sibling Relationships and Autism

**DOI:** 10.1007/s10803-024-06412-4

**Published:** 2024-06-11

**Authors:** Sebastian Trew

**Affiliations:** Institute of Child Protection Studies, Australian Catholic University, 223 Anthill Street, Canberra, Australian Capital Territory 2602 Australia

**Keywords:** Autism, Siblings, Families, Qualitative, Adolescence, Relationships

## Abstract

This study explores the experiences of siblings of autistic adolescents within families. Without the novel insights generated from engaging with siblings of autistic adolescents within a qualitative framework, it can be challenging to develop strategies for practicing effectively with this group or structuring interventions with these families. Using a social constructivist approach and qualitative participatory methodology, the exploratory study was conducted using in-depth interviews with ten non-autistic siblings, analyzed via a thematic analysis method. The results reveal a common feeling of uncertainty and apprehension in the relationships, leading siblings to physical and emotional detachment from their autistic brothers or sisters. The research emphasizes the caregiving roles predominantly taken up by sisters, especially when the autistic sibling is a brother with intellectual disability. These roles exert substantial demands, with unclear boundaries suggesting potential role confusion. The findings have important implications for family practice, necessitating the need to address role conflict and promote role clarity. They also underscore the gendered nature of caregiving, advocating for support to sister-siblings in these roles. This study revealed the complexities of sibling relationships in families with a member who is an autistic adolescent sibling. The study suggests interventions that promote open family dialogues for a balanced approach to family roles, providing valuable guidance to practitioners to enhance siblings and family well-being.

## Introduction

Autism spectrum disorder is a developmental condition thought to arise from a combination of complex, individualized genetic anomalies, and environmental factors (Abbeduto, 2019; Centre for Disease & Control Prevention, 2022). Though the diagnosis may be considered permanent, the symptoms and behaviours may fluctuate and include deficits in social communication and interaction and repetitive motor movements that impact on daily functioning (American Psychiatric Association, 2013). Autistic children can have difficulties developing and maintaining friendships, communicate with peers and adults as is socially expected, or have difficulty understanding the behaviors that are accepted from them in social situations including at school or at work (Centre for Disease & Control Prevention, 2022; Hyman et al., 2020).

Characteristic features of social communication and interaction encompass a range of behaviors. These include the avoidance of eye contact, a restricted range of facial expressions (e.g., happiness, sadness, anger), a lack of reciprocal interest sharing, reduced responsiveness to the emotional states of others (particularly in situations of distress or discomfort), and a notable absence of engagement in play or interactive activities with peers (American Psychiatric Association, 2013). Restricted and repetitive behavior include repetition of words or phrases known as echolalia, getting upset or stressed with changes to routine, obsessive interests, a need to follow a set routine, and stimming. Autistic people can also have sensory stimuli to sound, taste, smell, and touch. Sensory behaviours or needs can affect a person’s physical, emotional, relational, and social wellbeing, such as fear, anger, sadness, anxiety, and social isolation (Acker et al., 2018; American Psychiatric Association, 2013; Sibeoni et al., 2022; Spain et al., 2020). This may be coupled with a rigidity in behaviour, a need for persistent sameness in routine, and minimal social communication skills (Sibeoni et al., 2022).

Family members, including siblings, experience a wide range of mental health impacts when caring for an autistic family member (Australian Institute of Health & Welfare, 2019; Spain et al., 2020) and have a high level of needs for acquiring knowledge, identifying resources for social and emotional supports (Colombet et al., 2023). These are families who most likely experience challenges associated with reduced educational or social opportunities or respite and disability service support access and are known to experience difficulties and adversities due to a lack of these (D’Arcy et al., 2023). Parenting an autistic child has been described as difficult due to behaviours such as tantrums, aggression, and violence towards family members (Im, 2016). Grandparents of an autistic child can also be impacted (Prendiville & Kinsella, 2019) and include fewer familial interactions and less involvement in recreational activities than families who do not have a member who is an autistic person and when the presence of other social supports are limited (Spain et al., 2017; Trew, 2024). In some studies (Chan & Goh, 2013; Connell et al., 2014; Trew, 2021; Zechella & Raval, 2015) parents reported the impact autism had on the parent–child relationship with the child without an autism diagnosis. This included parent guilt due to a lack of time spent with their child and low levels of energy to engage meaningfully with them but also open and supportive relationship.

## Impacts on Siblings and Sibling Relationships

Reported impacts on siblings of an autistic brother or a sister in the family include stress and issues with anger, and the autistic siblings’ reduced communication and social interactions (Hartman, 2012;; Orsmond & Seltzer, 2007; Petalas et al., 2012). In a study investigating aggression and sibling interaction for siblings aged 8–15, 84% reported aggression as a key factor in interactions with their sibling with an autism diagnosis (Orsmond & Seltzer, 2007). In several studies (Mascha & Boucher, 2006; Ozsivadijan et al., 2012; Petalas et al., 2009; Ross & Cuskelly, 2006), communication and interaction deficits have been reported as a disappointment for siblings trying to interact with their autistic sibling, as the response reported in these studies was primarily aggression, violence, and unpredictable and erratic behaviour.

There are several studies that report on sibling relationship impacts when there is an autistic brother or sister in the family (Atkin & Tozer, 2014; Chu et al., 2021; Corsano et al., 2017; Cridland et al., 2016; Day et al., 2019; Diener et al., 2015; Gorjy et al., 2017; Hastings & Pet al.,as, 2013; Jones et al., 2019; Kaminsky & Dewey, 2002; Laghi et al., 2018; Nuttall et al., 2018; Orsmond & Fulford, 2018; Petalas et al., 2015; Petalas et al., 2009, 2012; Pollard, et al., 2013; Rivers & Stoneman, 2003; Roeyers & Mycke, 1995; Shivers & McGregor, 2019; Thomas et al., 2019; Tomeny et al., 2017; Tsai et al., 2018; Tudor et al., 2018; Walton et al., 2015). Most of these studies were conducted in the United States (US) and the United Kingdom and others were conducted in Canada, Italy, Belgium, Taiwan, and just two studies were conducted in Australia. Across the studies, informants were mostly older or adult siblings of autistic brothers, and some parents, mostly mothers. Just two of these studies (Petalas et al., 2015) a qualitative study conducted in the US and a qualitative study (Cridland et al., 2016) conducted in Australia included adolescent siblings as participants. There remain limited studies conducted in Australia with children and adolescents and from a qualitative perspective which have investigated autism and sibling relationship impacts.

In several of the studies (Laghi et al., 2018; Orsmond & Fulford, 2018; Pollard et al., 2013; Tomeny et al., 2017; Walton et al., 2015), negative outcomes found for the sibling relationship suggest, overall, negative interchanges between siblings and fewer positive sibling relationship attitudes than those where neither sibling has an autism diagnosis, including less involvement, and more avoidance. Three studies (Hastings & Pet al.,as, 2013; Jones et al., 2019) investigated behaviours and suggested that sibling relationships are at risk of poor outcomes when the autistic brother or sister has significant behavioral problems. Siblings across a few studies (Atkin & Tozer, 2014; Chu et al., 2021; Corsano et al., 2017; Cridland et al., 2016; Day et al., 2019; Gorjy et al., 2017; Tsai et al., 2018) reported on mixed experiences with their autistic sibling. These siblings shared the difficulty they experienced in juggling the changing and developing nature of their relationship with their autistic brother, describing relationships initially mixed with difficulty, sadness, and frustration at communication difficulties and the minimal exchange, then changing to encompassing love and protectiveness.

Some quantitative studies indicate that sibling relationship quality may be dependent on the level or severity of autism characteristics presenting in the diagnosed child (Petalas et al., 2009, 2012; Rivers & Stoneman, 2003). Other quantitative studies suggest that the birth order rank of the autistic child significantly moderates the relation between externalising behaviours in autistic children and externalising behaviours in their typically developing siblings, with higher levels of behaviour and conduct problems present in both only when the autistic child is the eldest (Orozco, 2014; Tomeny et al., 2014).

Quantitative studies have provided a firm understanding of the measurable impacts that autism has on siblings of autistic brothers and sisters. However, quantitative research does not fully investigate the effects and the variables on these relationships (Spain et al., 2017). This is a limitation in current knowledge because the “what is it like?” question is not addressed and the varying descriptions of human experiences within these groups are not revealed when quantitative measures are solely used (Wertz et al., 2011). As such, we do not have a good current understanding from a qualitative perspective of how and why autism impacts children and adolescent siblings and the relationship held with their autistic adolescent brother or sister.

## Research Aims and Question

Few studies within health psychology have qualitatively investigated family members’ perspectives on how autism impacts relationships between family members, such as partner or couple and spousal or marital dyad relationships, sibling dyad relationships, parent–child dyad relationships, and the family as a unit (Chan & Goh, 2013; Corsano et al., 2017; Cridland et al., 2016; D’Astous et al., 2013; Divan et al., 2012; McCabe, 2012; Petalas et al., 2015; Prendeville & Kinsella, 2019; Trew, 2021; Zechella & Raval, 2015). The qualitative studies that have investigated relationships in families with a member who is an autistic child have mostly focused on the parent or caregiver and their interactions with the autistic child. Many of these studies report understanding from a maternal perspective (Phelps et al., 2009; Smith et al., 2010) and limit the consideration of other perspectives or other familial relationships such as sibling relationships. A heavy focus on the maternal perspective might be because mothers are reported to be the primary caregiver of autistic children and young people (AIHW, 2019; ABS, 2016, 2020).

Although it is crucial to capture the maternal perspective, relying solely on this has limitations, as the maternal perspective may not be reflective of all family members, such as fathers or siblings or of autistic adolescents in the family. Without the perspectives of multiple family members there is limited guidance on how to structure family interventions that could offer mutual benefits and provide positive experiences for the family as a unit in a therapeutic setting, and limited guidance on how to identify issues specific to certain family member groups, such as non-autistic siblings (Varghese et al., 2020).

This study addresses this gap by including siblings of autistic brothers and sisters and provides some greater depth to the quantitative findings in the literature. Without the novel insights generated from engaging with siblings within a qualitative framework, it can be challenging to develop strategies for practicing effectively with this group or structuring interventions with these families.

In response to the gaps described above, this study seeks to investigate *how autism impacts the sibling relationship in families with an autistic adolescent family member.* To do so, it considers the perspectives of 10 siblings—four brothers and six sisters of autistic adolescents, drawn from 10 families. By doing so, this study aims to explore their perspectives concerning their experiences of the condition autism and the impact had on the relationships between them and the autistic adolescent sibling. The findings emphasize the importance of understanding siblings shared and diverse experiences in the context of living with and holding a relationship with their autistic brothers and sisters. Embedded throughout the findings, this study provides practice recommendations and guidance to practitioners working within families that help address the factors that influence the relationship experiences of siblings of autistic adolescents. This is to promote better well-being and quality of family-life for siblings.

## Research Design and Approach

This exploratory study is guided by a social constructivist approach, that personal construction of meaning is socially situated, experienced, and influenced by an individual’s interactions with others and their surrounding context (McKingley, 2015). Researchers working from a social constructivist approach typically rely on participant perspectives and do not aim to generalize to a group or population. The study utilizes a qualitative participatory methodology and was informed by a phenomenological approach to the research design. A phenomenological approach supports the research in departing from the natural sciences and focuses on the phenomena or lived experience (everyday world) of an individual (Gray, 2009). The participatory model adopted for this study acknowledged children and young people as active social agents in the telling of their *life worlds* (Davies, 2005). A participatory research methodology facilitated an approach to “join with” and “learn from” rather than “speak for” or “intervene into” children’s and young people’s lives (Cannella & Lincoln, 2011, p. 83). This framing helped to create an accessible space for children and adolescents to discuss and make sense of autism and their relationship experiences in their own ways. This meant remaining alert to the importance of keeping power balances in check and to ground the generation and development of themes in the narratives of the participants.

## Methods

Ethics approval for this study was provided by the Australian Catholic University National Human Research Ethics Committee, approval number 2019-33H. The study utilized data from interviews with 10 participants from 10 families who participated in the research. The study was conducted in the region of Canberra, Australian Capital Territory. Study information was advertised via local primary and high schools and disability support agencies. It was expected that around ten to twelve participants would be recruited and for data saturation to occur (Hennink et al., 2017; Luborsky & Rubinstein, 1995). Participants were 10 adolescents 15–18 years of age and comprised of six females and four males. Table 1 provides study participant information and further details of the participants and sampling plan are provided in Appendix A.

**Table 1:** Study participant information

Sibling	Age	Age of autistic brother/sister	Family
Sarah	15	17 (sister)	2 parent family
Layla	15	19 (sister with an ID)	2 parent family
Will	15	19 (brother)	2 parent family
Greg	15	19 (brother with an ID)	2 parent family
Martina	18	15 (brother)	2 parent family
Luke	18	16 (sister)	2 parent family
Chris	16	19 (sister)	2 parent family
Eileen	15	18 (brother with an ID)	2 parent family
Amandi	18	19 (brother with an ID)	Single parent family
Kate	18	19 (brother)	Single parent family

### Consent and Interview Process and Questions

Information about the research and project was provided to the study participants. Parental consent was required for all participants and a separate tick-box form was used to obtain the child's assent. Interviews were conducted in person with all participants using an interview guide and were audio recorded. Interviews were conducted with any sibling who expressed an interest in the study and who consented to an interview. Interviews ranged between 30–60 min. The interview guide listed broad open-ended semi-structured questions based on topics about siblings’ relationships, how they interacted and communicated as siblings, the positives, and strengths of the relationship and some of the challenges. Examples of questions asked of participants are provided in Appendix B.

### Data Analysis and Theme Construction

The data for analysis was the transcribed audio-recorded interviews and was analyzed using a thematic method, a qualitative approach known as thematic analysis (TA). Thematic analysis is a versatile method that can be applied to various epistemologies, interpretive frameworks, and research questions (Braun & Clarke, 2014; Charmaz, 2006). TA is employed to identify, analyze, organize, describe, and present themes derived from datasets (Braun & Clarke, 2006). In addition to providing a systematic structure for data, it allows for a comprehensive and in-depth investigation of the underlying patterns within the dataset (Attride-Stirling, 2001). The analysis process is provided in Appendix C.

### Reflexive Methods

Child and adolescent social research emphasize the use of reflexive methods and processes which highlight and focus on the role and position of the researcher (Bishop-Fitzpatrick & Rubenstein, 2019; James & Prout, 2015). To limit bias a reflexive stance was adopted throughout the research process (Attia & Edge, 2017). This included reflexive note taking and journaling to describe the researchers background, experiences, and perspective and to ensure the role of the researcher was documented as honestly as possible (Rice & Ezzy, 2007). When the role and position of the researcher are recognized and acknowledged, reflexivity is strengthened (Whittemore et al., 2001). Throughout the research process five main methods for reflexive practice were employed: (1) journaling, (2) critiquing interviews, (3) recording and written memos, (4) debriefing and supervision, (5) reflexive questioning during data analysis. These five main methods are described in Appendix D.

## Results

### Global Theme: Close Relationships Despite the Challenges

Siblings experienced a range of aggressive physical behaviours from their autistic brother or autistic sister such as kicking, punching, and hitting, and verbal behaviours, such as swearing and arguing. Common behavior that impacted the sibling relationship included overexcitement and minimal self-control, rudeness, and fixations or obsessions of the autistic sibling. Siblings shared that because of these kinds of behaviours, they found it “hard to cope” living with their autistic brother or autistic sister and needed time away isolated in their rooms. Despite the difficulties and challenges siblings faced, all siblings reported that they got along with or loved their autistic brother or autistic sister. Most siblings shared of how much they enjoyed hanging out and playing with their autistic brother or autistic sister.

Most older siblings and a few younger siblings shared their worries and concerns for a future spent caring for their autistic brother or autistic sister, particularly siblings who had an autistic brother or autistic sister with intellectual disability. Despite these concerns, most of these siblings experienced a relationship free from sibling rivalry or harassment. Notwithstanding the difficulties and challenges in the relationship, and siblings’ concerns about an ongoing caring role, all siblings expressed a deep love and care towards their autistic brother or autistic sister and reported getting along with them. Several subthemes were constructed within this global theme. These subthemes are presented and supported with anonymized participant quotes via pseudonyms.

### Subtheme 1: Getting Along

Within families, siblings appeared to be close with and protective of their autistic brother or autistic sister. Despite the difficult and challenging relationship dynamics within the sibling relationship, all siblings reported that they got along with or loved their autistic brother or autistic sister. For example, siblings Sarah said, “We get along”; and Martina said, “We like to get along a lot”. Luke reported, “I get along with them great”; and Amandi said, “Me and my brother, we love each other. We actually get along really well.”

Most siblings shared examples of how much they enjoyed hanging out and playing with their autistic brother or autistic sister. For example, sibling Layla said, “My sister is really funny, she can do a funny voice and it’s really funny and she says jokes and she plays around, it’s a lot of fun, I like playing with her like that.” Chris shared that “they are my favourite people in my family.”

In most families, the sibling dynamic fluctuated based on the behaviours of the autistic adolescent and the interactions between siblings. Families with multiple siblings, both autistic and non-autistic, generally characterized the sibling dynamic as a typical sibling relationship, regardless of autism being present. In most families with an autistic sibling with intellectual disability, the sibling relationship dynamic was typically described as close and loving. This was despite the minimally speaking interactions and reduced communications exhibited by the autistic sibling with intellectual disability. Sibling Amandi described the dynamic with her autistic brother with intellectual disability and reported on the absence of conflict in the relationship:

Amandi: [Our relationship] is a lot closer and has been for a lot longer than average. Longer than families if they didn’t have someone with autism and disability. We are really close as a result… A lot of friends throughout my life are absolutely at their siblings’ throats. They’ve always been complaining, “Oh, my sibling did this, they’re so annoying. We had this massive fight, this, that, and the other thing.” I stop and go I don’t know what that’s like. I don’t really know what it’s like to fight as siblings.

For the remaining families with an autistic brother or autistic sister with intellectual disability, sibling relationship dynamics were largely absent of conflict or rivalry.

### Subtheme 2: Not Just a Sibling but a Carer Too

Nearly half of all sibling study participants described a sibling relationship dynamic that differed from a typical sibling relationship. Greg reported that:

Greg: the neurotypical sibling often gets short-changed. They have to wear some of the burden of care of the person on the spectrum, if not directly supporting them. Yes, they’re a brother or sister, but indirectly in that way.

Families reported the relationship between siblings as strange, interesting, and different. Eileen described her relationship with her autistic brother as, “a bit of a strange relationship because I feel like when he does bad things, I also tell him not to do it even though he’s older than me.” Sister siblings described caregiving duties as a challenge to the sibling relationship. Amandi shared the challenges she experienced in caring for her autistic brother with intellectual disability:

Amandi: It’s probably I to think that it is a different relationship because it’s really not, because I am very much his carer. Challenges would be the fact that I do genuinely have to look after him. If I’m looking after him, I can’t just sit down and do my work and let him do his thing. I need to make sure he’s staying out of stuff. I need to make sure he gets fed.

Amandi further described her two roles in the relationship, one as a carer and one as a sister:

Amandi: I automatically shift into a caring role, but not in a bad way. Not like I’m just his carer and not his sister. I was happy to look after him, he’s, my autistic brother. I love him and from quite early on I knew how to look after him.

In most families with an autistic adolescent with intellectual disability, concern about sister siblings’ future care responsibilities for their autistic brother or sister weighed on some siblings’ minds. Eileen explained she wanted balance, seeking to have a “normal life” and see her autistic brother:

Eileen: I still want to be able to see [my autistic brother] a lot but also have a relatively normal life when I leave home would be good. I guess just being able to have my own place and grow up and do usual things and have kids and have a job. I’d like him to be part of my life, but I wouldn’t have to always be taking care of him.

### Subtheme 3: Aggression and Frustration

Nearly all siblings disclosed a range of behaviours that characterized the sibling relationship dynamic. All siblings reported their autistic brother or autistic sister kicking, punching, or hitting them, often accompanied by verbal outbursts, such as swearing. Noteworthy, no siblings reported any safety concerns and parents were reported to be responsive to the behavior.

Sarah explained: “Sometimes my sister, she’s angry, so sometimes I’m scared to say anything to her.” Kate shared that there are many times her autistic brother:

Kate: … will get frustrated at something, or something that [his other sister] does, or something that’s not quite going right, like he can’t get something to fit the way he wants it to, and they’ll be a bit of anger or frustration. I don’t want to deal with it, but we’re sort of working around that, “Maybe if we tried this, or if we tried that.” This is something that other siblings wouldn’t have to do without being forced really, to take other approaches.

Some siblings reported that autism played a significant role in the number of arguments present in the relationship. This affected how siblings felt about their autistic brother or autistic sister. Sarah reported:

Sarah: [My sister] gets too much sometimes. She has autism so she’s not always going to be like a normal sibling, because we have a lot of arguments. I reckon if she didn’t have as much, autism, like she didn’t really have autism, then I reckon we could definitely prevent more arguments. Because sometimes I really don’t like her, at the time.

Eileen said her sister can be, “really rude and mean, she swears at me a lot. When she’s being mean she’ll say very unnecessary things and kick and punch at me.” Sarah described physical and verbal conflict with her sister:

Sarah: He used to hit me and kick me a lot which I was kind of fine with, I just hit him back or fight him. He does a lot more verbal stuff now and I don’t like that, like swearing at me.

Sibling Will, describing his autistic brother as “difficult,” said that his autistic brother “jumps on me and attacks me and swears at me.” Some siblings reported that overwhelming stress led to meltdowns, escalations, frustrations, and self-harm. Amandi shared that her autistic brother, “can tell when someone loses their patience with him [due to stress], and he will escalate.” Several siblings reported on the impact of anger on the sibling relationship.

Most siblings reported a variety of irritating behaviours. For example, Kate said about her autistic brother: “He does things not because he’s trying to be annoying, and some of the things he does aren’t just because he’s a brother, it’s because he’s got a couple of things going on, autism is one.” Amandi reported on her need to automate her responses when stressed out by or interrupted by her autistic brother:

Amandi: I feel myself losing my patience, due to stress, or if he’s interrupting me when I am trying to work, I definitely lose my patience a lot. I really need to work on giving out automated responses without losing it.

### Subtheme 4: Time Together and Time Apart

Most siblings disclosed the negative impacts autism had on the time they spent together with their autistic brother or sister. For families with one sibling-dyad, the sibling dynamic was often described as being fraught with difficulty. In these families, siblings isolated themselves or needed time apart from their autistic brother or autistic sister. These siblings reported that the behaviour of their autistic brother or sister was the primary reason why they retreated. This subtheme shows how these behaviours are a reason why siblings want to separate from their autistic brothers and sisters.

Martina explained that she went to her room as a means of avoiding potential conflict with her autistic brother:

Martina: When [my autistic brother] is not in the right headspace it’s hard to talk to him and try to get him in the right headspace. I usually go to my room, so he doesn’t think that I’m a part of it.

Arguments between siblings were a main factor in the sibling dyad relationships that caused significant impacts on quality of time shared amongst siblings. Siblings had trouble coping with these arguments, which occurred daily. Some siblings felt torn, wanting to spend time with their autistic brother or autistic sister but feeling they had to stay away to avoid arguments. Sarah shared that she wanted to be able to spend more time peacefully with her autistic sister:

Sarah: I want to spend more time with my sister, like peacefully. I would be here on the couch doing things, but I’m afraid that my sister is going to say something to me, and I won’t either want to answer it, or feel the need to answer it. So that’s why I go to my room. I’m scared that she’s going to yell at me or do something. I know there’s going to be an argument.

Most siblings articulated the importance for service providers to help with arguments. Sarah shared that…:

Sarah: First, people could ask them [siblings] what it’s like at home for them, getting to know the family. I would ask them, are there many arguments [had with your autistic sibling] at home? … I would help with that. Because I know arguments are a really big thing in this house … like I said before, we cannot go one day without an argument.

A few siblings reported on erratic, repetitive, or obsessive behaviour that impacted on the quality of time that they spent together with their autistic brother. For some siblings, the repetitive or obsessive interests of the autistic brother limited the types of activities they could share and impacted the enjoyment for some autistic sisters. Eileen reported:

Eileen: I’ll do things with him like go on the trampoline. He’ll really want me to do it. … But it’s not that fun to go on the trampoline 50 times a day. Some things that he wants to do, I don’t necessarily want to do. Sometimes I do it and sometimes like, no!

Siblings across several families reported on the difficulty of understanding and connecting with their autistic brother or autistic sister. Siblings described ambiguity or uncertainty in the relationships with their autistic sibling. For example, Eileen said, “I guess I get along with my autistic brother. Sometimes, I don’t know, I just exist with him.” Kate reported, “I’ve struggled to connect with them … I still don’t quite get that perspective.” Amandi recalled the presence of her autistic brother whilst growing up, but the absence of a sibling playmate:

Amandi: Growing up I was like, “My autistic brother has a brain problem, so I don’t have anyone to play with at home.” Maybe I wouldn’t have felt so, not sure if alone is the right word, but isolated and like an outlier.

Supports identified for non-autistic siblings, such as sibling groups or mentors, were suggested by some siblings to help limit the factors of isolation and caring responsibilities in the sibling-dyad. For example, Amandi shared her idea for a sibling group with mentors:

Amandi: I think it would be really helpful. I wanted a siblings’ group because it’s very weird and I would have very much appreciated if someone, growing up, told me like, “Hey, it’s OK.” … Cause it’s very different talking to someone about caring who has no idea what’s going on, because they understand the condition [autism], but they don’t understand the actual experience of it, and that’s a game changer. … If someone was feeling isolated, I can help, I can be a sort of mentoring guide to someone who’s got a [an autistic] sibling. Because I never had anyone to talk to about that stuff growing up and so I wonder what would have changed…maybe I wouldn’t have felt so isolated and an outlier, it would be nice to have that sort of commonality between me and someone else.

## Discussion

The aim of this study was to explore with siblings of autistic adolescents their perspectives concerning their experiences of the condition autism. The key findings of the study: the disengagement and absence in the siblings’ relationships; and the unclear boundaries and role confusion observed in the siblings’ relationships, are now discussed and supported by theory.

### Disengagement and Absence in Sibling Relationships

Presented in subthemes 1, 3 and 4, a key finding from this study indicates potential disengagement and absence in the siblings’ relationships and the lack of attunement on the relationships. Disengagement observed in sibling relationships is characterized by emotional and physical withdrawal, alongside manifestations of hostility and anger (Bascoe et al., 2012). Such disengagement, indicative of a dismissing coping style, is associated with reduced relationship quality (Cichy et al., 2013). Within the family context, sibling disengagement manifests as aggression, rejection, and alienation, contributing to externalizing problems (Hetherington, 1999). Hetherington (1999) further describes these disengaged siblings as avoiding interaction, with their encounters marked by criticism and aggression. While acknowledging the limitations of applying Hetherington’s (1999) findings directly to the autism context, the study underscores the importance of cultivating effective coping strategies for siblings to manage stress and conflict in their relationships.

Attunement, or the capacity to recognize and respond to the needs and emotions of others, is crucial for relationship health (Perry, 2005; Trozzi & Dixon, 2006). However, the atypical behaviors and communication difficulties inherent in autism present significant challenges for siblings striving for attunement with their autistic siblings (Chang et al., 2018; Connor et al., 2020; Di Renzo et al., 2021). This study reveals that sisters often experience a psychological absence in their relationship with their autistic brothers, despite a physical presence, leading to feelings of loss and grief. This sense of absence is exacerbated by the autistic siblings’ restrictive and repetitive interests, highlighting a critical area for further investigation and support.

These experiences resonate with the concept of ambiguous loss, which describes a situation where there is a physical presence but a psychological absence, leading to unresolved grief (Boss, 1977, 1999, 2004, 2009). Originating from family stress theory, ambiguous loss theory suggests that the most profound family stresses are those that are unclear and difficult to resolve (Boss & Yeats, 2014; Carroll et al., 2007; Collings, 2008; Hillegas, 2012). This framework provides a valuable lens for understanding the unique challenges faced by siblings in families with an autistic sibling, emphasizing the need for supportive interventions that address these complex emotional landscapes.

### Unclear Boundaries and Role Confusion in Sibling Relationships

Presented in subthemes 2 and 3, this study reveals the intricate nature of sibling relationships affected by autism, marked by unclear boundaries and role confusion. The concept of boundaries, as defined by Berge and Holm (2007), interprets the expected roles and functions within a family, which can range from open to closed, impacting family cohesion and fluidity (Olson, 1985). In this context, the blurring of roles can lead to a sibling assuming additional responsibilities, often merging the roles of a sibling and a caregiver, especially prevalent among sisters of autistic brothers with an intellectual disability and in single parent families.

In instances where familial boundaries were ambiguous, there was an observed tendency for sisters to adopt caregiving or even parental roles, pointing towards a potential gendered aspect of caregiving in these family dynamics. This role expansion often led to a perceived vulnerability in their autistic brothers, whom they felt compelled to protect and support, despite the considerable impact on their own wellbeing.

The study aligns with the concept of boundary ambiguity described by Boss and Couden (2002) and Boss and Greenberg (1984), highlighting the confusion around family roles and membership. This ambiguity particularly affected sisters, who found themselves in a complex position of caregiving, compounded by the demands of emotional and physical support.

Sisters’ sense-making of the protective element of caring for autistic brothers helped these sisters to come to terms with the additional caregiving duties despite the significant impact these duties seemed to have on their wellbeing. The findings support previous research (Atkin & Tozer, 2014; Chu et al., 2021; Corsano et al., 2017; Day et al., 2019; Gorjy et al., 2017; Tsai et al., 2018) that reported on siblings mixed relational experiences with their autistic sibling. In these studies, and this study, siblings shared the difficulty they experienced in juggling the changing and developing nature of their relationship with their autistic brother, describing relationships initially mixed with difficulty, sadness, and frustration at communication difficulties and the minimal exchange, then changing to encompassing love and pride and protectiveness at great personal cost.

Furthermore, the study delves into family dynamics, particularly in single-parent households, where siblings may face increased responsibilities, altering the traditional sibling subsystem as suggested by family theory (Whiteman et al., 2011). This shift often places a significant emotional and logistical burden on the siblings, necessitating a broader support system. Attachment theory (Bowlby, 1969) suggests that the structure of social relationships and individual differences between a person and family members impacts their behavioral patterns (Whiteman et al., 2011). Attachment theory might help to explain how in these families the sibling may be engaged to care for their autistic brother or sister to ease demands on the parent or to serve as an ally with their parent against the aggressive behaviours of the autistic sibling (Drzymala et al., 2022).

However, family dynamics in a single-parent family might better explain these findings, too (Chanfreau & Goisis, 2024), as in the cases of the 2 siblings who lived without the father at home (due to divorce and death) the family dynamics are differentiated. This change in the family system has drastically altered the sibling’s subsystem- as the family theory suggests (Whiteman et al., 2011). In a single-parent family due to a loss (e.g., divorce or death) the relationships of the siblings and the roles assumed by the typically developing sibling towards the autistic sibling are differentiated.

The study findings also highlight the multifaceted nature of caregiving roles within these family structures, transcending simple household tasks to include emotional support and care, as outlined by Carpenter (2010) and Morris et al. (2007) and Schulz and Eden (2016). The study suggests that sisters of autistic brothers with intellectual disability took on a caregiving role in the sibling relationship and that this caregiver role was present despite the birth order, with some sisters being older and some being younger than their autistic brothers. This caregiving role, particularly pronounced in families with an autistic sibling with intellectual disabilities, underscores the need for a nuanced understanding of the challenges faced by these families.

### Implications for Practice

This study highlights the crucial role practitioners can play in supporting families of autistic adolescents, emphasizing the need to address the intricate dynamics and challenges these families face, particularly concerning role clarity and emotional well-being amongst siblings.

### Addressing Role Conflict and Emotional Well-Being

Role conflict and ambiguities within family structures can significantly contribute to psychological distress among family members, including depression and anxiety, a concern accentuated by the higher prevalence of these conditions in families caring for an autistic adolescent (Boss & Couden, 2002; Dababnah & Parish, 2016; Dabrowska & Pisula, 2010; Dale et al., 2006; Davis & Carter, 2008; Estes et al., 2009; Gray, 2003; Ludlow et al., 2011; Sharma et al., 2013; Spain et al., 2017). Practitioners can mitigate these risks by addressing role conflicts through family interventions, fostering an environment that encourages open discussion of each family member's roles and concerns and without attributing individual blame (Berge & Holm, 2007; Holm et al., 2008). For example, siblings could explore their concerns about their present and future role as carers without feelings of guilt as siblings often take on a caring role without preparation of planning (Long et al., 2023; Spain et al., 2017).

### Special Considerations for Sister Siblings

The study particularly underscores the unique position of sister siblings in families with an autistic brother with an intellectual disability, who often assume significant caregiving responsibilities highlighted by societal gender roles and familial needs in society which see females socialized to be nurturant and empathic (Connell et al., 2014; Cridland et al., 2016; Kaminsky & Dewey, 2002; Löffler & Greitemeyer, 2023; Long et al., 2023; Spain et al., 2017). Practitioners should be attuned to the gendered nature of caregiving, offering support and strategies to manage the increased caregiving load and pressures faced by sister siblings.

## Support Strategies and Family Interventions

Advocating for regular respite and facilitating access to resources and support services can alleviate the burden on sister siblings and the family. In single-parent families, this support becomes even more critical, given the amplified responsibilities placed on sister siblings. Family interventions that encourage a clear delineation of roles and responsibilities, guided by the concept of boundary ambiguity (Boss & Greenberg, 1984), can help in clarifying role expectations and reducing uncertainties. Such interventions aim to balance the boundaries within family relationships, ensuring a healthier dynamic and clearer role distinctions, especially between sibling and caregiver roles (Cridland et al., 2014).

## Conclusion and Study Limitations and Contributions

This explorative qualitative study has expanded the knowledge concerning how siblings, brothers and sisters of autistic adolescents, experience autism and the impacts had upon them and the relationship with the autistic sibling in the family. This study not just provides a voice to siblings but foregrounds their insights with theory and practice to provide suggestions and guidance for family therapists and practitioners when working with brothers and sisters of autistic adolescents and within families.

A limitation of this study is the small sample size and that the demographic profile of the study participants were mostly white heterosexual female and male participants. All participants were from middle to high socioeconomic status and from a single geographic area—being like other samples in previous studies investigating the sibling relationships in families with an autistic sibling member. Another limitation of the study is, it is possible that siblings and families experiencing greater difficulties, challenges, or impacts to their relationships than the ones reported on in this study, would not participate in this type of study. These are families who most likely experience challenges associated with reduced educational or social opportunities or respite and disability service support access. There is a recognised need for a greater focus on a more structured and thoughtful method for inquiring and efficiently responding to queries regarding siblings, their families, and impactful services for all involved (Tudor & Lerner, 2015).

Another limitation is that the study took the perspective of only one of the siblings and was based on self-reported data, which may lack some validity. These factors limit the generalizability of the study findings.

To conclude, a strength of the current study is that it reported on child and adolescent sister siblings who have an autistic brother or sister with intellectual disability, of which there are limited accounts reported in the literature. By doing so, the study provided novel insights into the experiences of adolescent sisters of autistic brothers with intellectual disability—a group whose voice would benefit further from the inclusion in future research on the topic. As such, this study offers tailored suggested guidance and practice for family therapists when working with or structuring an intervention with these adolescent sister siblings and their families.

## References

[CR1] Abbeduto, L., Thurman, A. J., McDuffie, A., Klusek, J., Feigles, R. T., Ted Brown, W., Harvey, D. J., Adayev, T., LaFauci, G., Dobkins, C., & Roberts, J. E. (2019). ASD comorbidity in Fragile X syndrome: Symptom profile and predictors of symptom severity in adolescent and young adult males. *Journal of Autism and Developmental Disorders,**49*(3), 960–977. 10.1007/s10803-018-3796-2 30382442 10.1007/s10803-018-3796-2 PMC6556533

[CR2] Acker, L., Knight, M., & Knott, F. (2018). ‘Are they just gonna reject me?’ Male adolescents with autism making sense of anxiety: An interpretative phenomenological analysis. *Research in Autism Spectrum Disorders,**56*, 9–20.

[CR3] American Psychiatric Association, DSM-5 Task Force. (2013). *Diagnostic and statistical manual of mental disorders: DSM-5™* (5th ed.). American Psychiatric Publishing Inc., Amsterdam. 10.1176/appi.books.9780890425596

[CR4] Atkin, K., & Tozer, R. (2014). Personalisation, family relationships and autism: Conceptualising the role of adult siblings. *Journal of Social Work,**14*(3), 225–242. 10.1177/1468017313476453

[CR5] Attia, M., & Edge, J. (2017). Be(com)ing a reflexive researcher: A developmental approach to research methodology. *Open Review of Educational Research,**4*(1), 33–45. 10.1080/23265507.2017.1300068

[CR6] Attride-Stirling, J. (2001). Thematic networks: An analytic tool for qualitative research. *Qualitative Research,**1*(3), 385–405. 10.1177/146879410100100307

[CR7] Australian Bureau of Statistics. (2016). *Autism in Australia*. https://www.abs.gov.au/ausstats/abs@.nsf/mf/4428.0

[CR8] Australian Institute of Health and Welfare. (2019). *Autism in Australia*. https://www.aihw.gov.au/reports/disability/autism-in-australia/contents/autism

[CR9] Australian Bureau of Statistics. (2020). *Autism in Australia. Disability, Ageing and Carers, Australia: Summary of Findings*. https://www.abs.gov.au/ausstats/abs@.nsf/Lookup/4430.0Main%20Features752015

[CR10] Bascoe, S. M., Davies, P. T., & Cummings, E. M. (2012). Beyond warmth and conflict: The developmental utility of a boundary conceptualization of sibling relationship processes. *Child Development,**83*(6), 2121–2138. 10.1111/j.1467-8624.2012.01817.x 22862542 10.1111/j.1467-8624.2012.01817.x PMC3500585

[CR11] Berge, J., & Holm, K. (2007). Boundary ambiguity in parents with chronically ill children: Integrating theory and research. *Family Relations,**56*, 123–134. 10.1111/j.1741-3729.2007.00446.x

[CR12] Bishop-Fitzpatrick, L., & Rubenstein, E. (2019). The physical and mental health of middle aged and older adults on the autism spectrum and the impact of intellectual disability. *Research in Autism Spectrum Disorder,**63*, 34–41. 10.1016/j.rasd.2019.01.001 10.1016/j.rasd.2019.01.001 PMC6876625 31768189

[CR13] Boss, P. (1977). A clarification of the concept of psychological father presence in families experiencing ambiguity of boundary. *Journal of Marriage and the Family,**39*(1), 141–151. 10.2307/351070

[CR14] Boss, P. (1999). *Ambiguous loss*. Harvard University Press.

[CR15] Boss, P. (2004). Ambiguous loss research, theory, and practice: Reflections after 9/11. *Journal of Marriage and Family,**66*(3), 551–566. 10.1111/j.0022-2445.2004.00037.x

[CR16] Boss, P. (2006). *Loss, trauma, and resilience: Therapeutic work with ambiguous loss*. W Norton & Co.

[CR17] Boss, P. (2009). The trauma and complicated grief of ambiguous loss. *Pastoral Psychology,**59*(2), 137–145. 10.2307/351070

[CR18] Boss, P., & Couden, B. A. (2002). Ambiguous loss from chronic physical illness: Clinical interventions with individuals, couples, and families. *Journal of Clinical Psychology,**58*(11), 1351–1360. 10.2307/351070 12412146 10.1002/jclp.10083

[CR19] Boss, P., & Greenberg, J. (1984). Family boundary ambiguity: A new variable in family stress theory. *Family Process,**23*(4), 535–546. 10.1111/j.1545-5300.1984.00535.x 6519247 10.1111/j.1545-5300.1984.00535.x

[CR20] Boss, P., & Yeats, J. (2014). Ambiguous loss: A complicated type of grief when loved ones disappear. *Bereavement Care,**33*, 63–69. 10.2307/35107010.1080/02682621.2014.933573

[CR21] Bowlby, J. (1969). *Attachment and loss* (Vol. 1). Basic Books.

[CR22] Braun, V., & Clarke, V. (2006). Using thematic analysis in psychology. *Qualitative Research in Psychology,**3*(2), 77–101. 10.1191/1478088706qp063oa

[CR23] Braun, V., & Clarke, V. (2014). What can “thematic analysis” offer health and wellbeing researchers? *International Journal of Qualitative Studies on Health and Well-Being,**9*(1), 1–2. 10.3402/qhw.v9.26152 10.3402/qhw.v9.26152 PMC4201665 25326092

[CR24] Cannella, G., & Lincoln, Y. (2011). *Ethics, research regulations, and critical social science*. The Sage Handbook of Qualitative Research.

[CR25] Carpenter, B. E., & Mulligan, A. (2010). Assessment with late-life families: Issues and instruments. In A. L. Peter (Ed.), *Handbook of assessment in clinical gerontology* (2nd ed., pp. 273–304). New York: Academic Press. 10.1016/B978-0-12-374961-1.10011-9

[CR26] Carroll, J. S., Olson, C. D., & Buckmiller, N. (2007). Family boundary ambiguity: A 30-year review of theory, research, and measurement. *Family Relations,**56*(2), 210–230. 10.1111/j.1741-3729.2007.00453.x

[CR27] Centre for Disease and Control Prevention. (2022). Autism Spectrum Disorder. https://www.cdc.gov/ncbddd/autism/index.html

[CR28] Chan, G. W., & Goh, E. C. (2013). “My parents told us that they will always treat my brother differently because he is autistic”—Are siblings of autistic children the forgotten ones? *Journal of Social Work Practice,**28*(2), 155–171. 10.1080/02650533.2013.844114

[CR29] Chanfreau, J., & Goisis, A. (2024). Patterns of help and care by adult only children and children with siblings. *Ageing and Society,**44*(1), 200–223. 10.1017/S0144686X22000198

[CR30] Chang, Y.-C., Shih, W., Landa, R., Kaiser, A., & Kasari, C. (2018). Symbolic play in school-aged minimally verbal children with autism spectrum disorder. *Journal of Autism and Developmental. Disorders,**48*, 1436–1445. 10.1007/s10803-017-3388-6 29170936 10.1007/s10803-017-3388-6

[CR31] Charmaz, K. (2006). *Constructing grounded theory: A practical guide through qualitative analysis*. SAGE Publications.

[CR32] Chu, Y. S., Kassim, S. N. Z., Gan, C. H., et al. (2021). “Sometimes I Feel Grateful…”: Experiences of the Adolescent Siblings of Children with Autism Spectrum Disorder in Malaysia. *Journal of Autism and Devlopmental Disorders*. 10.1007/s10803-021-05184-5 10.1007/s10803-021-05184-5 PMC8272616 34247302

[CR33] Cichy, K., Lefkowitz, E., Griffin, E., & Fingerman, K. (2013). “You are such a disappointment!”: Negative emotions and parents’ perceptions of adult children’s lack of success. *The Journals of Gerontology Series b, Psychological Sciences and Social Sciences,**68*(6), 893–901. 10.1093/geronb/gbt053 23733857 10.1093/geronb/gbt053 PMC3805291

[CR34] Collings, C. (2008). That’s not my child anymore! Parental grief after acquired brain injury (ABI): Incidence, nature and longevity. *The British Journal of Social Work,**38*, 1499–1517. 10.1093/bjsw/bcm055

[CR35] Colombet, C., Alcaraz, C., de la Tribonnière, X., Morsa, M., & Rattaz, C. (2023). Self-reported needs of caregivers of people with Autism Spectrum Disorder. *Journal of Autism and Developmental Disorders,**53*, 2798–2805. 10.1007/s10803-022-05499-x 35441919 10.1007/s10803-022-05499-x

[CR36] Conner, C. M., White, S. W., Scahill, L., & Mazefsky, C. (2020). The role of emotion regulation and core autism symptoms in the experience of anxiety in autism. *Autism,**24*, 931–940. 10.1177/1362361320904217 32050774 10.1177/1362361320904217 PMC7773149

[CR37] Corsano, P., Musetti, A., Guidotti, L., & Capelli, F. (2017). Typically developing adolescents’ experience of growing up with a brother with an autism spectrum disorder. *Journal of Intellectual & Developmental Disability,**42*(2), 151–161. 10.3109/13668250.2016.1226277

[CR38] Creswell, J. W., & Poth, C. N. (2018). *Qualitative inquiry and research design: Choosing among five approaches*. Sage.

[CR39] Cridland, E. K., Jones, S. C., Stoyles, G., Caputi, P., & Magee, C. A. (2016). Families living with autism spectrum disorder: Roles and responsibilities of adolescent sisters. *Focus on Autism and Other Developmental Disabilities,**31*(3), 196–207. 10.1177/1088357615583466

[CR40] Cridland, L., Jones, S., Caputi, P., & Magee, C. (2014). Qualitative research with families living with autism spectrum disorder: Recommendations for conducting semistructured interviews. *Journal of Intellectual and Developmental Disability,**40*, 78–91. 10.3109/13668250.2014.964191

[CR41] D’Arcy, E., Burnett, T., Capstick, E., Elder, C., Slee, O., Girdler, S., Scott, M., & Milbourn, B. (2023). The well-being and support needs of Australian caregivers of neurodiverse children. *Journal of Autism and Developmental Disorders*. 10.1007/s10803-023-05910-1 36757543 10.1007/s10803-023-05910-1 PMC9909132

[CR42] D’Astous, V., Wright, S. D., Wright, A. C., & Diener, M. L. (2013). Grandparents of grandchildren with autism spectrum disorder: Influences on engagement. *Journal of Intergenerational Relationships,**11*(2), 134–147. 10.1080/15350770.2013.782744

[CR43] Dababnah, S., & Parish, S. L. (2016). Feasibility of an empirically based program for parents of preschoolers with autism spectrum disorder. *Autism,**20*(1), 85–95. 10.1177/1362361314568900 25717131 10.1177/1362361314568900

[CR44] Dabrowska, A., & Pisula, E. (2010). Parenting stress and coping styles in mothers and fathers of pre-school children with autism and Down syndrome. *Journal of Intellectual Disability Research,**54*(3), 266–280. 10.1111/j.1365-2788.2010.01258.x 20146741 10.1111/j.1365-2788.2010.01258.x

[CR45] Dale, E., Jahoda, A., & Knott, F. (2006). Mothers’ attributions following their child’s diagnosis of autistic spectrum disorder. *Autism,**10*(5), 463–479. 10.1177/1362361306066600 16940313 10.1177/1362361306066600

[CR46] Davies, B. (2005). Communities of practice: Legitimacy not choice. *Journal of Sociolinguistics,**9*, 557–581. 10.1111/j.1360-6441.2005.00306.x

[CR47] Davis, N. O., & Carter, A. S. (2008). Parenting stress in mothers and fathers of toddlers with autism spectrum disorder: Associations with child characteristics. *Journal of Autism and Developmental Disorders,**38*(7), 1278–1291. 10.1007/s10803-007-0512-z 18240012 10.1007/s10803-007-0512-z

[CR48] Day, A., Johner, R., Chalmers, D., & Novik, N. (2019). Sibling relationships and autism spectrum disorder: A different relationship. *Canadian Social Work,**20*(2), 88–107.

[CR49] Di Renzo, M., Guerriero, V., Pagnacco, A., Petrillo, M., Racinaro, L., D’Errico, S., & Bianchi di Castelbianco, F. (2021). Attunement and paternal characteristics in care relationships in the presence of children diagnosed with autism. *International Journal of Environmental Research and Public Health*. 10.3390/ijerph18042010 33669600 10.3390/ijerph18042010 PMC7922537

[CR50] Diener, M. L., Anderson, L., Wright, C. A., & Dunn, L. M. (2015). Sibling relationships of children with autism spectrum disorder in the context of everyday life and a strength-based program. *Journal of Child and Family Studies,**24*, 1060–1072. 10.1007/s10826-014-9915-6

[CR51] Divan, G., Vajaratkar, V., Desai, M. U., Strik-Lievers, L., & Patel, V. (2012). Challenges, coping strategies, and unmet needs of families with a child with autism spectrum disorder in Goa, India. *Autism Research: Official Journal of the International Society for Autism Research,**5*(3), 190–200. 10.1002/aur.1225 22473816 10.1002/aur.1225 PMC4991760

[CR52] Drzymala, H., Grey, B., & Fowler, N. (2023). Exploring the triadic parent–child–sibling relationship: How do mothers’ view of their children impact sibling relationships? *Human Systems: Therapy, Culture and Attachments,**3*(2), 92–111. 10.1177/26344041221145619

[CR53] Estes, A., Munson, J., Dawson, G., Koehler, E., Zhou, X., & Abbott, R. (2009). Parenting stress and psychological functioning among mothers of preschool children with autism and developmental delay. *Autism,**13*(4), 375–387. 10.1177/1362361309105658 19535467 10.1177/1362361309105658 PMC2965631

[CR54] Gibbs, G. (2018). *Analyzing qualitative data (2nd ed.).* Sage Publications Ltd. https://www.doi.org/10.4135/9781526441867

[CR55] Gorjy, R., Fielding, A., & Falkmer, M. (2017). “It’s better than it used to be”: Perspectives of adolescent siblings of children with an autism spectrum condition. *Child & Family Social Work*. 10.1111/cfs.12371

[CR56] Gray, D. E. (2003). Gender and coping: The parents of children with high functioning autism. *Social Science & Medicine,**56*(3), 631–642. 10.1016/s0277-9536(02)00059-x 12570979 10.1016/s0277-9536(02)00059-x

[CR57] Gray, D. E. (2009). *Doing research in the real world*. Sage.

[CR58] Hartmann, A. (2012). Autism and its impact on families [Unpublished Masters of Social Work dissertation]. Sophia, the St. Catherine University repository website: https://sophia.stkate.edu/msw_papers/35

[CR59] Hastings, R. P., & Petalas, M. A. (2013). Self-reported behaviour problems and sibling relationship quality by siblings of children with autism spectrum disorder. *Child: Care, Health and Development,**40*(6), 833–839. 10.1111/cch.12131 10.1111/cch.12131 24460897

[CR60] Hennink, M. M., Kaiser, B. N., & Marconi, V. C. (2017). Code saturation versus meaning saturation: how many interviews are enough? *Qualitative Health Research,**27*(4), 591–608. 10.1177/1049732316665344 27670770 10.1177/1049732316665344 PMC9359070

[CR61] Hetherington, E. (1999). Family functioning and the adjustment of adolescent siblings in diverse types of families. *Monographs of the Society for Research in Child Development, 64*(4), 1–25. http://www.jstor.org/stable/3181537 10.1111/1540-5834.00045 10685435

[CR62] Hillegas, E. (2012). *Family experiences of ambiguous loss in response to serious childhood illness: Parental perspectives*. Social Work Master’s Clinical Research Papers. 45. https://ir.stthomas.edu/ssw_mstrp/45

[CR63] Holm, K. E., Patterson, J. M., Rueter, M. A., & Wamboldt, F. (2008). The impact of uncertainty associated with a child’s chronic health condition on parents’ health. *Families, Systems & Health: THe Journal of Collaborative Family Healthcare,**26*(3), 282–295. 10.1037/a0012912 10.1037/a0012912 PMC2873603 20490375

[CR64] Hyman, S. L., Levy, S. E., Myers, S. M., AAP Council on Children with Disabilities, Section on developmental and behavioral pediatrics. (2020). Identification, evaluation, and management of children with autism spectrum disorder. *Pediatrics,**145*(1), e20193447. 31843864 10.1542/peds.2019-3447

[CR65] Im, D. S. (2016). Template to perpetrate: An update on violence in autism spectrum disorder. *Harvard Review of Psychiatry,**24*(1), 14–35. 10.1097/HRP.0000000000000087 26735321 10.1097/HRP.0000000000000087 PMC4710161

[CR66] James, A., Jenks, C., & Prout, A. (2015). *Theorizing childhood*. Polity Press.

[CR67] Jones, E. A., Fiani, T., & Stewart, J. L. (2019). When one sibling has autism: Adjustment and sibling relationship. *Journal of Child and Family Studies,**28*, 1272–1282. 10.1007/s10826-019-01374-z

[CR68] Kaminsky, L., & Dewey, D. (2002). Psychosocial adjustment in siblings of children with autism. *Journal of Child Psychology and Psychiatry,**43*(2), 225–232. 10.1111/1469-7610.00015 11902601 10.1111/1469-7610.00015

[CR69] Laghi, F., Lonigro, A., Pallini, S., et al. (2018). Sibling relationships and family functioning in siblings of early adolescents, adolescents and young adults with autism spectrum disorder. *Journal of Child and Family Studies,**27*, 793–801. 10.1007/s10826-017-0921-3

[CR70] Long, K. A., LaRochelle, J., Gordillo, M., Pariseau, E. M., DeCelle, M. G., & Orsmond, G. (2023). Siblings FORWARD: Development of a new program to engage siblings of autistic adults in future planning. *Journal of Autism and Developmental Disorders*. 10.1007/s10803-023-06178-1 38117420 10.1007/s10803-023-06178-1

[CR71] Luborsky, M. R., & Rubinstein, R. L. (1995). Sampling in qualitative research: Rationale, issues, and methods. *Research on Aging,**17*(1), 89–113. 10.1177/0164027595171005 22058580 10.1177/0164027595171005 PMC3207270

[CR72] Ludlow, A., Skelly, C., & Rohleder, P. (2011). Challenges faced by parents of children diagnosed with autism spectrum disorder. *Journal of Health Psychology,**17*(5), 702–711. 10.1177/1359105311422955 22021276 10.1177/1359105311422955

[CR73] Mascha, K., & Boucher, J. (2006). Preliminary investigation of a qualitative method of examining siblings’ experiences of living with a child with ASD. *The British Journal of Development Disabilities,**52*(102), 19–28. 10.1179/096979506799103659

[CR74] McCabe, H., & Barnes, R. E. (2012). Autism in a family in China: An investigation and ethical consideration of sibling issues. *International Journal of Disability, Development and Education,**59*(2), 197–207. 10.1080/1034912X.2012.676440

[CR75] McKinley, J. (2015). Critical argument and writer identity: Social constructivism as a theoretical framework for EFL academic writing. *Critical Inquiry in Language Studies,**12*(3), 184–207.

[CR76] Morris, A. S., Silk, J. S., Steinberg, L., Myers, S. S., & Robinson, L. R. (2007). The role of the family context in the development of emotion regulation. *Social Development,**16*(2), 361–388. 10.1111/j.1467-9507.2007.00389.x 19756175 10.1111/j.1467-9507.2007.00389.x PMC2743505

[CR77] Nuttall, A. K., Coberly, B., & Diesel, S. J. (2018). Childhood caregiving roles, perceptions of benefits, and future caregiving intentions among typically developing adult siblings of individuals with autism spectrum disorder. *Journal of Autism and Developmental Disorders,**48*(4), 1199–1209. 10.1007/s10803-018-3464-6 29368234 10.1007/s10803-018-3464-6

[CR78] Oktay, J. S. (2012). Grounded theory. *Oxford Scholarship Online*. 10.1093/acprof:oso/9780199753697.001.000

[CR79] Olson, D. H., Portner, J., & Lavee, Y. (1985). *Faces III: Family adaptability & cohesion evaluation scales, family social science*. Minnesota: University of Minnesota.

[CR80] Orozco, T. (2014). *"Impact Of Birth Order On Autism Spectrum Disorder Children’s Typically Developing Siblings"*. Electronic Theses, Projects, and Dissertations. 116. https://scholarworks.lib.csusb.edu/etd/116

[CR81] Orsmond, G. I., & Fulford, D. (2018). Adult siblings who have a brother or sister with autism: Between-family and within-family variations in sibling relationships. *Journal of Autism and Developmental Disorders,**48*(12), 4090–4102. 10.1007/s10803-018-3669-8 29971655 10.1007/s10803-018-3669-8 PMC7386436

[CR82] Orsmond, G. I., & Seltzer, M. M. (2007). Siblings of individuals with autism spectrum disorders across the life course. *Mental Retardation and Developmental Disabilities Research Reviews,**13*(4), 313–320. 10.1002/mrdd.20171 17979200 10.1002/mrdd.20171

[CR83] Ozsivadjian, A., Knott, F., & Magiati, I. (2012). Parent and child perspectives on the nature of anxiety in children and young people with autism spectrum disorders: A focus group study. *Autism,**16*(2), 107–121. 10.1177/1362361311431703 22297200 10.1177/1362361311431703

[CR84] Perry, S. (2005). In Encyclopedia of Mental Health (Third Edition). Editors Howard S. Friedman, Charlotte H. Markey, Academic Press, 2023, Pages 292–302, 10.1016/B978-0-323-91497-0.00212-5.

[CR85] Petalas, M., Hastings, R., Nash, S., & Duff, S. (2015). Typicality and subtle difference in sibling relationships: Experiences of adolescents with autism. *Journal of Child and Family Studies,**24*, 38–49. 10.1007/s10826-013-9811-5

[CR86] Petalas, M. A., Hastings, R. P., Nash, S., Lloyd, T., & Dowey, A. (2009). Emotional and behavioural adjustment in siblings of children with intellectual disability with and without autism. *Autism,**13*(5), 471–483. 10.1177/1362361309335721 19759062 10.1177/1362361309335721

[CR87] Petalas, M. A., Hastings, R. P., Nash, S., Reilly, D., & Dowey, A. (2012). The perceptions and experiences of adolescent siblings who have a brother with autism spectrum disorder. *Journal of Intellectual & Developmental Disability,**37*(4), 303–314. 10.3109/13668250.2012.734603 23171311 10.3109/13668250.2012.734603

[CR88] Phelps, K. W., McCammon, S. L., Wuensch, K. L., & Golden, J. A. (2009). Enrichment, stress, and growth from parenting an individual with an autism spectrum disorder. *Journal of Intellectual & Developmental Disability,**34*(2), 133–141. 10.1080/13668250902845236 19404834 10.1080/13668250902845236

[CR89] Pollard, C. A., Barry, C. M., Freedman, B. H., et al. (2013). Relationship quality as a moderator of anxiety in siblings of children diagnosed with autism spectrum disorders or down syndrome. *Journal of Child and Family Studies,**22*, 647–657. 10.1007/s10826-012-9618-9

[CR90] Prendeville, P., & Kinsella, W. (2019). The role of grandparents in supporting families of children with autism spectrum disorders: A family systems approach. *Journal of Autism and Developmental Disorders,**49*, 738–749. 10.1007/s10803-018-3753-0 30229360 10.1007/s10803-018-3753-0

[CR91] Rice, P., & Ezzy, D. (2007). *Qualitative research methods: A health focus*. Masako Ono-Kihara. https://eprints.utas.edu.au/5006/

[CR92] Roeyers, H., & Mycke, K. (1995). Siblings of a child with autism, with mental retardation and with a normal development. *Child: Care, Health and Development,**21*(5), 305–319. 10.1111/j.1365-2214.1995.tb00760.x 8529293 10.1111/j.1365-2214.1995.tb00760.x

[CR93] Ross, P., & Cuskelly, M. (2006). Adjustment, sibling problems and coping strategies of brothers and sisters of children with autistic spectrum disorder. *Journal of Intellectual & Developmental Disability,**31*(2), 77–86. 10.1080/13668250600710864 16782592 10.1080/13668250600710864

[CR94] Saldaña, J. (2009). *The coding manual for qualitative researchers*. Sage Publications Ltd.

[CR95] Schulz, R., & Eden, J. (2016). *Families caring for an aging America*. Berlin: National Academies Press. 27905704

[CR96] Sharma, S., Winter, D., & McCarthy, M. (2013). A personal construct approach to understanding stress in mothers of children diagnosed with autism spectrum disorders. *Journal of Constructivist Psychology,**26*(1), 50–61. 10.1080/10720537.2013.732534

[CR97] Shivers, C. M., & McGregor, C. M. (2019). Brief report: Sibling feelings toward their brother or sister with or without autism or intellectual disability. *Journal of Autism and Developmental Disorders,**49*(1), 404–409. 10.1007/s10803-018-3694-7 30043352 10.1007/s10803-018-3694-7

[CR98] Sibeoni, J., Massoutier, L., Valette, M., Manolios, E., Verneuil, L., Speranza, M., & Revah-Levy, A. (2022). The sensory experiences of autistic people: A metasynthesis. *Autism*. 10.1177/13623613221081188 35362340 10.1177/13623613221081188

[CR99] Smith, J., Flowers, P., & Larkin, M. (2009). *Interpretative phenomenological analysis: Theory, method and research*. Sage.

[CR100] Smith, T. (2010). Early and intensive behavioral intervention in autism. In J. R. Weisz & A. E. Kazdin (Eds.), *Evidence-based psychotherapies for children and adolescents* (pp. 312–326). The Guilford Press.

[CR101] Spain, D., Sin, J., Paliokosta, E., Furuta, M., Prunty, J. E., Chalder, T., Murphy, D. G., & Happé, F. G. (2017). Family therapy for autism spectrum disorder. *Cochrane Database of Systematic Reviews,**5*(5), 11894. 10.1002/14651858.CD011894.pub2 10.1002/14651858.CD011894.pub2 PMC6484452 28509404

[CR102] Spain, D., Zıvralı Yarar, E., & Happé, F. (2020). Social anxiety in adults with autism: A qualitative study. *International Journal of Qualitative Studies on Health and Well-Being,**15*(1), 1803669. 32815779 10.1080/17482631.2020.1803669 PMC7482774

[CR103] Thomas, B. R., Lafasakis, M., & Spector, V. (2019). A child with autism spectrum disorder teaches siblings to skateboard: Effects on sibling skills and family social behavior. *Child & Family Behavior Therapy,**41*(3), 125–140. 10.1080/07317107.2019.1635391

[CR104] Tomeny, T. S., Barry, T. D., & Bader, S. H. (2014). Birth order rank as a moderator of the relation between behavior problems among children with an autism spectrum disorder and their siblings. *Autism: the International Journal of Research and Practice,**18*(2), 199–202. 10.1177/1362361312458185 23045222 10.1177/1362361312458185

[CR105] Tomeny, T. S., Ellis, B. M., Rankin, J. A., & Barry, T. D. (2017). Sibling relationship quality and psychosocial outcomes among adult siblings of individuals with autism spectrum disorder and individuals with intellectual disability without autism. *Research in Developmental Disabilities,**62*, 104–114. 10.1016/j.ridd.2017.01.008 28122290 10.1016/j.ridd.2017.01.008

[CR115] Trew, S. (2021). Family relationships and autism spectrum disorder: Lived experiences of young people with autism and their families. School of Allied Health, Faculty of Health Sciences, Australian Catholic University. https://acuresearchbank.acu.edu.au/item/8y446/family-relationships-and-autism-spectrum-disorder-lived-experiences-of-young-people-with-autism-and-their-families

[CR117] Trew, S. (2024). Made to feel different: Families perspectives on external responses to autism and the impacts on family wellbeing and relationships. *Autism*. 10.1177/13623613231221684 10.1177/13623613231221684 PMC11301965 38240288

[CR106] Trozzi, M & Dixon, S.D. (2006). Chapter 13 - Eight to Nine Months: Exploring and Clinging, Editor(s): Suzanne D. Dixon, Martin T. Stein, Encounters with Children (Fourth Edition), Mosby, 2006, Pages 292–321, 10.1016/B0-32-302915-9/50017-3.

[CR107] Tsai, H. J., Cebula, K., Liang, S. H., & Fletcher-Watson, S. (2018). Siblings’ experiences of growing up with children with autism in Taiwan and the United Kingdom. *Research in Developmental Disabilities,**83*, 206–216. 10.1016/j.ridd.2018.09.001 30248583 10.1016/j.ridd.2018.09.001

[CR108] Tudor, M. E., & Lerner, M. D. (2015). Intervention and support for siblings of youth with developmental disabilities: A systematic review. *Clinical Child and Family Psychology Review,**18*, 1–23. 10.1007/s10567-014-0175-1 25315924 10.1007/s10567-014-0175-1

[CR109] Tudor, M. E., Rankin, J., & Lerner, M. D. (2018). A model of family and child functioning in siblings of youth with autism spectrum disorder. *Journal of Autism and Developmental Disorders,**48*(4), 1210–1227. 10.1007/s10803-017-3352-5 29067588 10.1007/s10803-017-3352-5

[CR110] Varghese, M., Kirpekar, V., & Loganathan, S. (2020). Family interventions: Basic principles and techniques. *Indian Journal of Psychiatry*, *Suppl S2*, 192–200. https://www.indianjpsychiatry.org/text.asp?2020/62/8/192/276097 10.4103/psychiatry.IndianJPsychiatry_770_19 PMC7001353 32055062

[CR111] Walton, K. M., & Ingersoll, B. R. (2015). Psychosocial adjustment and sibling relationships in siblings of children with autism spectrum disorder: Risk and protective factors. *Journal of Autism and Developmental Disorders,**45*(9), 2764–2778. 10.1007/s10803-015-2440-7 25847756 10.1007/s10803-015-2440-7

[CR112] Wertz, F., Charmaz, K., McMullen, L.M., Josselson, R., Anderson, R., & McSpadden, E. (2011). Five ways of doing qualitative analysis: Phenomenological psychology, grounded theory, discourse analysis, narrative research, and intuitive inquiry. Guildford Press.

[CR113] Whiteman, S. D., McHale, S. M., & Soli, A. (2011). Theoretical perspectives on sibling relationships. *Journal of Family Theory & Review,**3*(2), 124–139. 10.1111/j.1756-2589.2011.00087.x 21731581 10.1111/j.1756-2589.2011.00087.x PMC3127252

[CR114] Whittemore, R., Chase, S. K., & Mandle, C. K. (2001). Validity in qualitative research. *Qualitative Health Research,**11*(4), 522–537. 10.1177/104973201129119299 11521609 10.1177/104973201129119299

[CR116] Zechella, A., & Raval, V. (2015). Parenting children with intellectual and developmental disabilities in Asian Indian families in the United States. *Journal of Child and Family Studies,**25*, 1295–1309. 10.1007/s10826-015-0285-5

